# Early use of airway pressure release ventilation in acute respiratory distress syndrome induced by coronavirus disease 2019: a case report

**DOI:** 10.1186/s13256-022-03658-3

**Published:** 2022-12-27

**Authors:** Jadeny Sinatra, Ronnie Wirawan Salim, Epifanus Arie Tanoto, Hori Hariyanto

**Affiliations:** 1 Department of Anesthesiology, Faculty of Medicine, Universitas Methodist Indonesia, Medan, Sumatera Utara Indonesia; 2 Anesthesiology Department, Siloam Dhirga Surya Hospital, Medan, Sumatera Utara Indonesia; 3 Emergency Department, Siloam Dhirga Surya Hospital, Medan, Sumatera Utara Indonesia; 4 Anesthesiology Department and Critical Care Medicine, Siloam Hospital Lippo Village, Tangerang, Banten, Indonesia

**Keywords:** Airway pressure release ventilation, COVID-19, Acute respiratory distress syndrome, Case report

## Abstract

**Background:**

Coronavirus disease 2019 is a highly transmissible and pathogenic viral infection caused by severe acute respiratory syndrome coronavirus 2, a novel coronavirus that was identified in early January 2020 in Wuhan, China, and has become a pandemic disease worldwide. The symptoms of coronavirus disease 2019 range from asymptomatic to severe respiratory failure. In moderate and severe cases, oxygen therapy is needed. In severe cases, high-flow nasal cannula, noninvasive ventilation, and invasive mechanical ventilation are needed. Many ventilation methods in mechanical ventilation can be used, but not all are suitable for coronavirus disease 2019 patients. Airway pressure release ventilation, which is one of the mechanical ventilation methods, can be considered for patients with moderate-to-severe acute respiratory distress syndrome. It was found that oxygenation in the airway pressure release ventilation method was better than in the conventional method. How about airway pressure release ventilation in coronavirus disease 2019 patients? We report a case of confirmed coronavirus disease 2019 in which airway pressure release ventilation mode was used.

**Case presentation:**

In this case study, we report a 74-year-old Chinese with a history of hypertension and uncontrolled diabetes mellitus type 2. He came to our hospital with the chief complaint of difficulty in breathing. He was fully awake with an oxygen saturation of 82% on room air. The patient was admitted and diagnosed with severe coronavirus disease 2019, and he was given a nonrebreathing mask at 15 L per minute, and oxygen saturation went back to 95%. After a few hours with a nonrebreathing mask, his condition worsened. On the third day after admission, saturation went down despite using noninvasive ventilation. We decided to intubate the patient and used airway pressure release ventilation mode. Finally, after 14 days of being intubated, the patient could be extubated and discharged after 45 days of hospitalization.

**Conclusion:**

Early use of airway pressure release ventilation may be considered as one of the ventilation strategies to treat severe coronavirus disease 2019 acute respiratory distress syndrome. Although reports on airway pressure release ventilation and protocols on its initiation and titration methods are limited, it may be worthwhile to consider, given its known ability to maximize alveolar recruitment, preserve alveolar epithelial integrity, and surfactant, all of which are crucial for handling the “fragile” lungs of coronavirus disease 2019 patients.

## Introduction

A new disease emerged in early January 2020 in Wuhan, China, caused by a novel ribonucleic acid (RNA) coronavirus, named coronavirus disease 2019 (COVID-19) by the World Health Organization (WHO) and has become a pandemic disease worldwide [1, 2]. Up to the end of July 2021, the WHO reported that COVID-19 had caused 4 million deaths and infected more than 194 million individuals worldwide. [3]

Severe acute respiratory syndrome coronavirus-2 (SARS-CoV-2) enters the body through the angiotensin-converting enzyme-2 (ACE-2) receptors, followed by intracellular translocation. ACE-2 receptors are found in abundance within the lower respiratory tract, which explains the high incidence of cough. Disease severity can be highly variable, ranging from asymptomatic to acute respiratory distress syndrome (ARDS) and fulminant multiorgan failure. Other clinical presentations include pyrexia, fatigue, anosmia, myalgia, sore throat, headache, diarrhea, and dyspnea. These variations may be related to the route of transmission, inoculation dose, and host immunity status [4].

The median duration of ARDS to intubation from the first symptoms developing is 8 days [5, 6]. Male gender, older age, and underlying comorbidities such as hypertension, diabetes, cardiovascular disease, and cerebrovascular disease are associated with a higher mortality rate [4, 5, 7, 8]. The severity of ARDS at admission also increases the in-hospital mortality rate [8]. Management of COVID-19 ARDS (CARDS) depends on the phenotype. For the type 2 or type “H” phenotype, a patient with severe hypoxemia, reduced pulmonary compliance, high lung elastance, high lung weight, and high lung recruit ability, the lung protective ventilation strategy should be used, which includes low tidal volume (LTV) (6 mL/kg ideal body weight), low positive end expiratory pressure (PEEP; 10 cmH_2_O), and fraction of inspired oxygen (FiO_2_) levels as tolerated to avoid poor tissue perfusion and plateau pressure (30 cmH_2_O) [9] However, some authors also use higher PEEP (10–15 cmH_2_O) in the type 2 phenotype [4]. The following ventilation strategies represent an expert opinion; therefore, further data is required to confirm the efficacy of the strategies. Airway pressure release ventilation (APRV) may be considered early for intubated patients with moderate-to-severe ARDS to provide adequate alveolar recruitment, but many providers are still not familiar with this ventilation mode. [8] In this case study, we report the use of APRV in CARDS.

## Case presentation

In our case, a 74-year-old Chinese male presented with the chief complaint of feeling fatigue, headache, nausea, and loss of appetite for 7 days after the first symptom developed. The patient had a history of type 2 diabetes mellitus and hypertension, and routinely consumed metformin-glibenclamide and amlodipine. There is no smoking and no alcohol consumption history. The patient had not received the vaccine as it was still not available at that time. Upon initial assessment, the patient was fully awake with a Glasgow Coma Scale (GCS) of 15, and the patient did not feel dyspnea despite his peripheral oxygen saturation (SpO_2_) being 82% and his respiratory rate of 22 breaths per minute. His blood pressure was 174/86 mmHg with 95 pulses per minute, and his body temperature was 37.3 °C. On physical examination, the patient was tachypneic without using respiratory muscles, and there was no sign of neurological deficit.

The patient’s laboratory results on admission were positive for lymphocytopenia, abnormal transaminase, hyponatremia, and uncontrolled diabetes, with high C-reactive protein (CRP) and ferritin, and a ratio of arterial oxygen partial pressure to fraction of inspired oxygen (P/F ratio) showing severe acute respiratory distress. The patient’s laboratory results are presented in Table 1.

**Table 1:** Laboratory tests on admission

Laboratory measurement	Patient result	Reference range
Hemoglobin (Hb)	16.4	11.7–15.5 (g/dL)
White blood cells	8.27	4.0–10.0 (10^3^/µl)
Neutrophil count	7.11	1.5–7.0 (10^3^/µl)
Lymphocyte count	0.48	1.00–3.70 (10^3^/µl)
Platelet	162	150–400 (10^3^/µl)
Urea	39.3	16.6–49.5 (mg/dL)
Creatinine	1.06	0.51 –0.95 (mg/dL)
Alanine serum transaminase	56	< 33 (U/L)
Aspartate serum transaminase	56	< 32 (U/L)
Ferritin	1999	12–300 (ng/mL)
D-Dimer	0.24	0–0.5 (µg/mL)
C-reactive protein (CRP)	57	< 6 (mg/dL)
HbA_1_C	8.0	< 7 (%)
Sodium	126	135–155 (mEq/L)
Potassium	3.4	3.5–5.3 (mEq/L)
Chloride	89	94–111 (mEq/L)
P/F ratio	77	mmHg

*HbA1c* Glycated Hemoglobin A1c Antibody;* P/F ratio* Ratio of arterial oxygen partial pressure to fraction of inspired oxygen

**Fig. 1:**
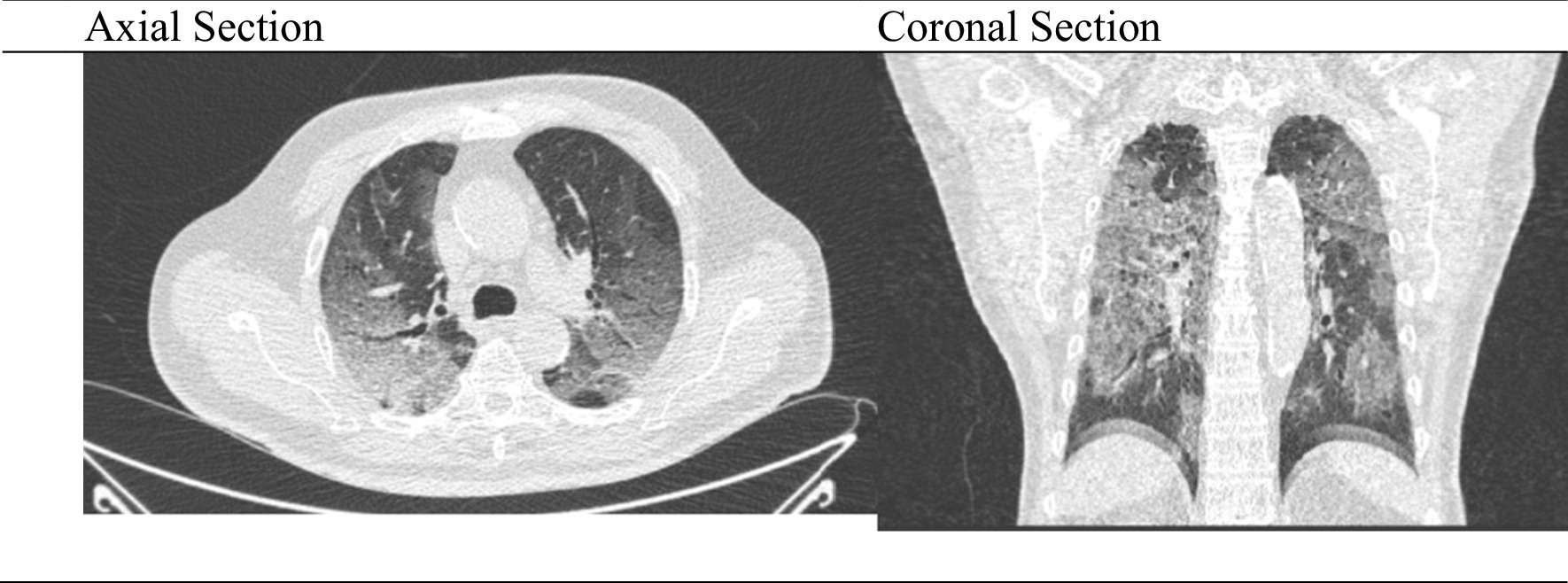
Image of the thorax computerized tomography scan axial and coronal section on admission

The patient also underwent a chest computerized tomography (CT) scan, and ground glass opacity was found on both lungs, suggestive of viral pneumonia (Fig. 1). The polymerase chain reaction (PCR) for COVID-19 were positive. The patient was diagnosed with severe COVID-19, with hypertension and diabetes mellitus as comorbidities.

The patient was treated with the following: oxygen (15 L per minute) using a nonrebreathing mask (NRM), and oxygen saturation improved to 95%; lopinavir–ritonavir combination with oral hydroxychloroquine as per the antiviral treatment guideline at the time; intravenous dexamethasone 6 mg once daily for 10 days; heparin 5000 units subcutaneous once daily; intravenous omeprazole 40 mg twice daily; intravenous acetaminophen 1 g three times daily; oral supplements such as zinc, vitamin E, and vitamin C 500 mg intravenously three times daily; intravenous azithromycin 500 mg once daily; and intravenous meropenem 1 g three times daily for prophylactic antibiotic. For the comorbidities he received oral candesartan 8 mg once daily, subcutaneous (SC) preprandial insulin three times daily and SC detemir insulin at night.

After 6 hours on the NRM, the patient became tachypneic with a respiratory rate of 30 breaths per minute, and oxygen saturation dropped to 90%; hence, he was treated with a high-flow nasal cannula (HFNC). The next day, his condition worsened with a respiratory rate of 35 breaths per minute and a drop in SpO_2_ to 88%. The patient was then treated with noninvasive ventilation (NIV) in continuous positive airway pressure (CPAP) mode and was sedated with midazolam and morphine. The patient was also treated with tocilizumab on this day. On the third day, the patient desaturated to 77% and blood gas analysis showed pO_2_ 44 mmHg, pCO_2_ 64 mmHg with FiO_2_ 100% despite using NIV. He was then intubated with the APRV method with the following settings: P high 28, P low 0, T high 5 seconds, T low 0.5 seconds, FiO_2_ 90%, and the patient was intubated for 14 days. The patient’s condition was evaluated every 24 hours with serial arterial blood gas analysis. During APRV mode, the patient was noted to be comfortable with no ventilator asynchrony. The weaning process was evaluated by the P/F ratio. First, the weaning process was done by lowering the FiO_2_ step by step until FiO_2_ reached 40%, then the P high was lowered by 2 cmH_2_O and the release pressure ventilation was lowered to 1–2 per minute. After P high was lowered to 18 cmH_2_O and FiO_2_ was 40%, the ventilation mode was changed to adaptive support ventilation (ASV).

**Fig. 2:**
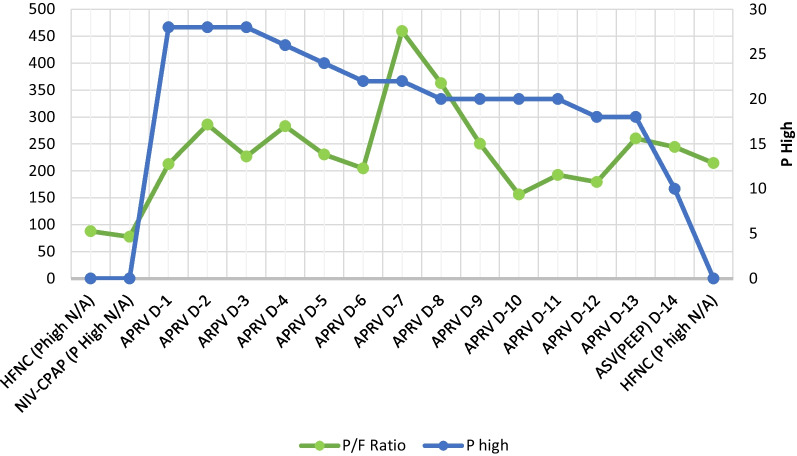
The airway pressure release ventilation settings and the P/F ratio. The P/F ratio was low at admission, but after using airway pressure release ventilation, the P/F ratio progressively increases

**Fig. 3:**
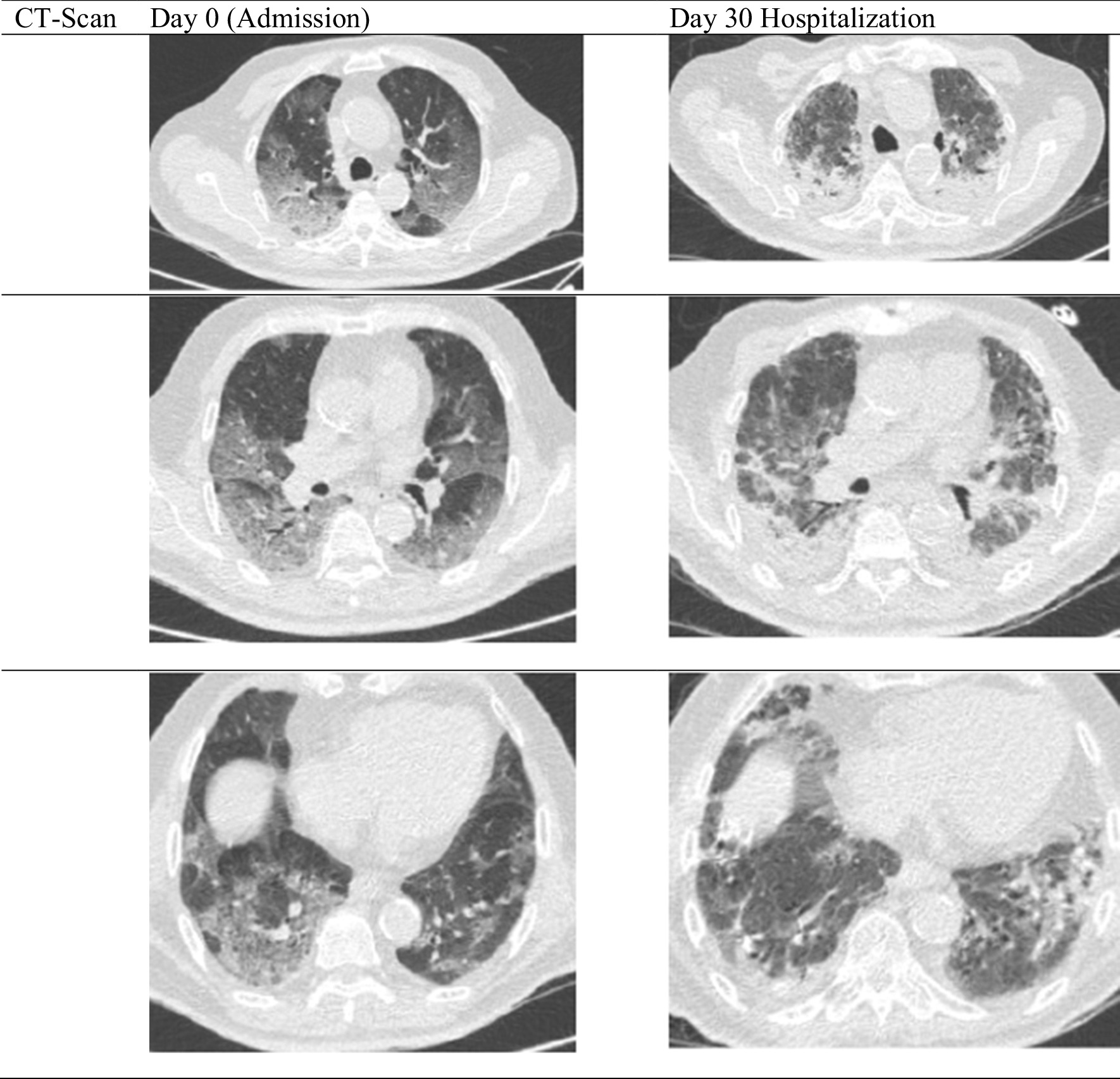
Thoracal computerized tomography scan comparison between the first admission day and day 30 hospitalization

On the first day of APRV, the patient’s blood was evaluated and showed leukocyte increased to 16.66 × 10^3/^µl, with d-dimer of 2.82 µg/mL, ferritin > 2000 ng/mL, CRP 34 mg/dL, and procalcitonin 0.45 ng/mL, hence the patient was given additional medication including fluconazole 200 mg every 12 hours, 20 g intravenous immunoglobulin (IVIG) once daily for 5 days, and a continuous heparin drip at 250 units per hour, Azithromycin and meropenem were continued. On the third day of APRV, the patient was evaluated again and the blood results showed that leukocytes had increased to 17.87 × 10^3^/µl and d-dimer was 2.62 µg/mL. Azithromycin and meropenem were changed to intravenous cefoperazone sulbactam 2 g twice daily and intravenous levofloxacin 750 mg daily. On the fifth day of APRV, after 10 days of dexamethasone, the dexamethasone was stopped. On the sixth day of APRV, the patient had thick, yellow and bloody sputum. The heparin drip was discontinued, and the patient had a sputum culture. The next day, fluconazole was stopped and changed to micafungin 100 mg once daily as the patient’s blood tests showed that aspartate aminotransferase (AST) and alanine aminotransferase (ALT) rose to 282 U/L and 230 U/L, respectively, while leukocytes were still at 17.14 × 10^3/^µl, d-dimer lowered to 1.57 µg/mL, procalcitonin was 0.07 ng/mL, and renal function test was normal. On the ninth day of APRV, the patient’s full blood count was evaluated, and leukocytes had increased to 21.23 × 10^3^/µl; cefoperazone sulbactam and levofloxacin were changed to intravenous tigecycline 100 mg loading dose then 50 mg twice daily and intravenous moxifloxacin 400 mg once daily. *Pseudomonas luteola* was found on the sputum culture on the 13th day of APRV, with intermediate sensitivity to tigecycline. Hence, tigecycline was changed to intravenous piperacillin/tazobactam 4.5 g four times daily, while quinolone was sensitive. The patient was then weaned to ASV and the next day was extubated and weaned until HFNC. The patient was weaned day by day from HFNC until he did not require oxygen supplementation. On the 30th day of hospitalization, the patient’s lung was re-examined using a thoracic CT scan, shown in Figs. 2 and 3. The patient was discharged after 45 days of hospitalization. One year follow-up showed that the patient was in a healthy condition, can work and is active and independent.

The APRV settings and P/F ratio when intubated are shown in Fig. 2. During NIV-CPAP with pressure support of 10 cmH_2_O, the patient’s P/F ratio was 77. On day 1 after intubation, when P high was set at 28 cmH_2_O, the P/F ratio was 213, which is an improvement from severe ARDS to mild ARDS.

## Discussion

Our patient was admitted with severe COVID-19, and his condition worsened and he desaturated to 77% despite oxygen supplementation using NIV-CPAP. When a patient is intubated, a conventional mode of ventilation with an LTV strategy is usually used. However, we used the early APRV mode of ventilation , which outperformed the conventional method in terms of oxygenation, ventilator-free days, and length of stay in the intensive care unit (ICU).

APRV is one of the ventilation modes that was introduced in 1987. APRV is a pressure-controlled, intermittent mandatory ventilation, applied using inverse ratio ventilation. The mandatory breaths applied are time triggered, pressure targeted, and time cycled (depending on the ventilator, trigger and cycle events may be synchronized with patient breathing signals). Spontaneous breathing can occur both during and between mandatory breaths [10]. The purpose of inverse ratio ventilation in APRV is to provide a shortened expiratory phase to permit an adequate tidal volume to escape without allowing alveoli to fall below their closing volume [11].

APRV is considered an “open lung approach” to mechanical ventilation [12]. On the pressure–volume curve, the lower inflection point (LIP) represents the initial point at which alveoli are readily recruited, and below the LIP alveoli tend to collapse. The upper inflection point (UIP) represents the point at which alveoli become overdistented [10, 11]. In APRV, the amplitude of the time-triggered mandatory breath is called “P high” instead of inspiratory pressure, and the duration when pressure is applied is called “T high” instead of inspiratory time. The expiratory pressure is called “P low” and the release time, or expiratory time, is called “T low” [10].

The P high was set below the UIP and the P low was usually set at 0 cmH_2_O because of the development of auto-PEEP using APRV mode. This auto-PEEP maintains the airway pressure above the LIP on the pressure–volume curve. By keeping the P high and P low between the two inflection points, the tidal volume received by the patient is the most compliant portion of the curves. Because P high is set by the operator, the potential for ventilator-induced lung injury is minimized [11].

APRV improves alveolar recruitment by its long-term high and constant airway pressure, which maximizes alveolar recruitment and promotes collateral ventilation through the pores of Kohn [11]. The ability to trigger spontaneous breathing in ARPV reduces asynchronization with the ventilator, thus improving patient comfort and reducing the need for sedation and neuromuscular blocking agents. Reduced sedation may reduce the incidence of constipation, cardiovascular depression, and cough reflex depression, all of which contribute to clearance and lower the risk of ventilator-associated pneumonia (VAP) [10]. Using a neuromuscular blocking agent can cause polyneuropathy. Because of spontaneous breathing during APRV, diaphragm muscle atrophy caused by prolonged mechanical ventilation can be prevented [11]. Greater hemodynamic performance can be seen in APRV because of spontaneous breathing. Decreased intrathoracic pressure during inspiration augments systemic venous return to the heart from the abdominal organs, hence improving cardiac output [13].

There have been multiple experiments in animal models in which APRV improves arterial oxygenation, increases ventilation in dependent areas of the lung, reduces inflammatory cytokine production, and can prevent the development of ARDS [14]. In a porcine model with sepsis-induced and ischemia/reperfusion-induced lung injury, experiments compared the effectiveness of APRV in preventing ARDS with that of low tidal volume mechanical ventilation. APRV was applied to animals 1 hour after sepsis was induced, while LTV was applied to animals when the criteria for mild ARDS were met (P/F ratio of 300). The study found that APRV prevented clinical and histological lung injury by preserving alveolar epithelial integrity, reducing lung edema, preserving surfactant, and maintaining alveolar stability [15]. In a rat model with pulmonary ARDS, APRV was compared with volume-controlled ventilation; alveolar overdistention was seen more in the volume-controlled ventilation group than in APRV. In the APRV group, there was less expression of amphiregulin, a gene that is expressed during times of alveolar stretch [11].

Nevertheless, using APRV mode does have its downside owing to its long T high, which can induce hypercapnia. More importantly, the degree of hypercapnia and respiratory acidosis tolerated by each patient varies. Some groups are not tolerant of hypercapnic conditions, such as those with coronary artery disease, arrhythmias, pulmonary hypertension, right ventricular dysfunction, and brain injury [14].

Until now, there have been no guidelines on the optimal APRV settings and titration strategy. However, there are two specific protocols proposed by Habashi and Zhou. In the Habashi protocol, P high was set at the desired plateau pressure, typically between 20–35 cmH_2_O, P low was set at 0 cmH2O, T high was set at 4–6 seconds, and T low was set at 0.2–0.8 seconds for restrictive lung disease and 0.8–1.5 seconds for obstructive lung disease. The PEEP was set at no more than 0 cmH_2_O because the airway resistance would create auto-PEEP [11]. The weaning process begins if the FiO_2_ is 40% and the SpO_2_ is 95%. The P high was lowered and the T high was increased [11].While, according to the Zhou protocol, the P high was set at no more than 30 cmH2O, the P low was set at 5 cmH_2_O, and the T low was set at 1–1.5 times the expiratory time constant, and then adjusted to achieve termination of peak expiratory flow rate (PEFR) of more than 50% PEFR, the release frequency was 10–14 times per minute, and the T high was indirectly calculated based on the T low and release frequency [13].

As in our case report, the patient was treated with P high at 28 cmH_2_O, which if the P high was converted from the volume-cycled mode, is the plateau pressure or peak airway pressure in the pressure-cycled mode. The *P*-value was set at zero to allow end-expiratory or released lung volume to be controlled by time only. The inherent resistance of the artificial airway behaves as a flow resistor and, if coupled with a brief release time, can create auto-PEEP [12].

Zhout *et al*. compared the early use of APRV mode with low tidal volume mechanical ventilation in ARDS. APRV was found to improve oxygenation significantly on the third day, with a higher P/F ratio in the APRV group. In this case, our patient showed improved oxygenation and a higher P/F ratio on the first day after intubation [13]. Based on the Carsetti *et al*. meta-analysis comparing APRV with conventional ventilation strategies in patients with acute hypoxemic failure, APRV has a higher number of ventilator-free days, at 28; a lower intensive care unit (ICU) length of stay; lower hospital mortality; and a higher mean arterial pressure than conventional ventilation [16].

## Conclusion

In our case, using APRV in elderly patients with uncontrolled diabetes mellitus, hypertension, and severe COVID-19 ARDS could improve the P/F ratio from the first day of using APRV. Early use of APRV may be considered one of the ventilation strategies to treat severe COVID-19 ARDS. Although reports on APRV and protocols on its initiation and titration methods are limited, it may be worthwhile to consider, given its known ability to maximize alveolar recruitment and preserve alveolar epithelial integrity and surfactant, all of which are crucial for handling the “fragile” lungs of COVID-19 patients.

## Data Availability

Please contact Jadeny Sinatra for Data Requests.

## Abbreviations

ACE -2: Angiotensin-converting enzyme 2
APRV: Airway pressure release ventilation
ARDS: Acute respiratory distress syndrome
ASV: Adaptive support ventilation
CARDS: COVID-19 acute respiratory distress syndrome
COVID-19: Corona virus disease 2019
CPAP: Continuous positive airway pressure
CRP: C-reactive protein
CT: Computerized tomography
FiO_2_: Fraction of inspired oxygen
HFNC: High-flow nasal cannula
I: Intra venous
IVIG: Intravenous immunoglobulin
LIP: Lower inflection point
LTV: Low tidal volume
NIV: Noninvasive ventilation
NRM: Nonrebreathing mask
P/F Ratio: Ratio of arterial oxygen partial pressure to fraction of inspired oxygen
PEEP: Positive end expiratory pressure
PEFR: Peak expiratory flow rate
RNA: Ribonucleic acid
SARS CoV2: Severe acute respiratory syndrome coronavirus-2
SC: Subcutaneous
SpO_2_: Peripheral oxygen saturation
UIP: Upper inflection point
VAP: Ventilator-associated pneumonia
WHO: World Health Organization
